# Evaluating disruption scenarios for improving downstream oil supply chain resilience and cost minimization using Monte Carlo simulation

**DOI:** 10.1038/s41598-025-22678-9

**Published:** 2025-11-06

**Authors:** Malik Asad Hayat Awan, Afshan Naseem, Ali Ahsan, Asjad Shahzad, Mehran Ullah, Ali Safaa Sadiq

**Affiliations:** 1 Department of Engineering Management, College of Electrical and Mechanical Engineering, National University of Sciences and Technology, Islamabad, Pakistan; 2 Torrens University Australia, Adelaide, Australia; 3 School of Business and Creative Industries, University of the West of the Scotland, Paisley, PA1 2BE Scotland, UK; 4 Department of Computer Science, Nottingham Trent University, Clifton Lane, Nottingham, NG11 8NS UK

**Keywords:** Downstream oil, Supply chain optimization, Heuristic search algorithms, Monte carlo simulation, Applied mathematics, Computational science, Statistics

## Abstract

This study examines the impact of both random and anticipated disruptions on transportation costs within different stages of a downstream oil supply chain. Conducting a comprehensive literature review, a MILP model was developed to simulate a multifaceted refined oil supply chain, integrating refining and import facilities, storage depots, and customer demand nodes. The study unfolds in two phases: a deterministic model establishing a supply chain performance baseline, and a Monte Carlo simulation generating disruption scenarios. Results reveal increased transportation costs and significant flow modifications between entities. Imports of refined oil products surged to counter local production shortages, with increased use of cost-effective bulk cargo modes and a notable reliance on road transport to offset disrupted pipelines. The study highlights the substantial impact of disruptions on transportation costs, emphasizing diversified transportation methods where pipelines are constrained. Acknowledging study limitations focusing on a singular supply chain’s transport costs, it advocates for research on inventory management and alternate pipeline development to enhance supply chain resilience under disruption scenarios.

## Introduction

Today, the oil industry operates one of the most advanced and intricate supply chains globally. As per the Energy Institute’s report, the global energy consumption in 2022 soared to an impressive 52,969 TWh, solidifying oil’s status as the most coveted commodity of the 21 st century. Operating as a quintessential supply chain, the petroleum industry seamlessly integrates supply facilities and manufacturing centers across the upstream, midstream, and downstream segments. Disruptions within this complex network can arise from various sources, encompassing technical failures, emergencies, supply issues, and natural calamities, ultimately impacting functions, as highlighted by Azad et al.^1^. Additionally, the disruptive impact of the COVID-19 pandemic was keenly felt as it impeded the supply of crude oil to refineries, underscoring the critical need for diversified sourcing and robust contract management as vital mitigation strategies^2^. The aftermath of the Ukraine-Russia war has also resulted in the crude oil prices to become rise significantly that have impacted global energy supply chains^3^.

The downstream landscape is characterized by a complex interplay of refineries, distribution centers, transportation infrastructure, and diverse refined product demands, necessitating a strategic approach to network design. The distribution of goods between downstream facilities is separated into two categories: primary distribution and secondary distribution. The primary is responsible for distributing oil products across refineries, petrochemical plants, and depots in the wholesale segment. It originates in the refineries and ends at transshipment points. The principal distribution modes are pipeline (pipelines), marine (ships), railroad (train wagons), and road (tank trucks). The secondary transportation starts in storage depots and moves oil products from domestic, international, and regional petrochemical plants and refineries to the end consumers in the retail sector or exports them abroad. Tank trucks and occasionally railway wagons are the transportation methods that are most frequently utilized in secondary distribution. Strategic decisions encompass optimizing distribution center location, capacity, and interlinkages, while tactical considerations involve regulating product flows, managing inventory, and selecting appropriate transport modes. To ensure efficient and seamless operations, companies must strategically source refined products from multiple refineries and import terminals, aligning transportation quantities with facility capacities and demand nodes.

Worldwide instability has resulted in disruptions becoming increasingly unpredictable as well as more frequent in all kinds of industries. The modern-day petroleum and oil supply chain (POSC) is vertically integrated, covering all segments from exploration and refining, all the way to distribution at fuel stations. The entire supply chain can be separated into three parts namely upstream, midstream, and downstream. The POSC is extremely rigid as well as complex, while also proving to be a significant risk with a high impact on the economies of the world. The complications lead to the formation of risk of various types that need to be measured when designing, developing and operating such a system^4^. The war in Ukraine has severely impacted the global logistics, which has increased the pressure on already overused and stressed systems, ultimately resulting in widespread disruption of services^5^. The primary reasons are the sanctions by Western Europe along with closure of the sea route from the Black Sea into the Mediterranean, thereby restricting exports from Russia. It can be said that the most worthwhile illustration of this scenario is crop supplies.

**Fig. 1:**
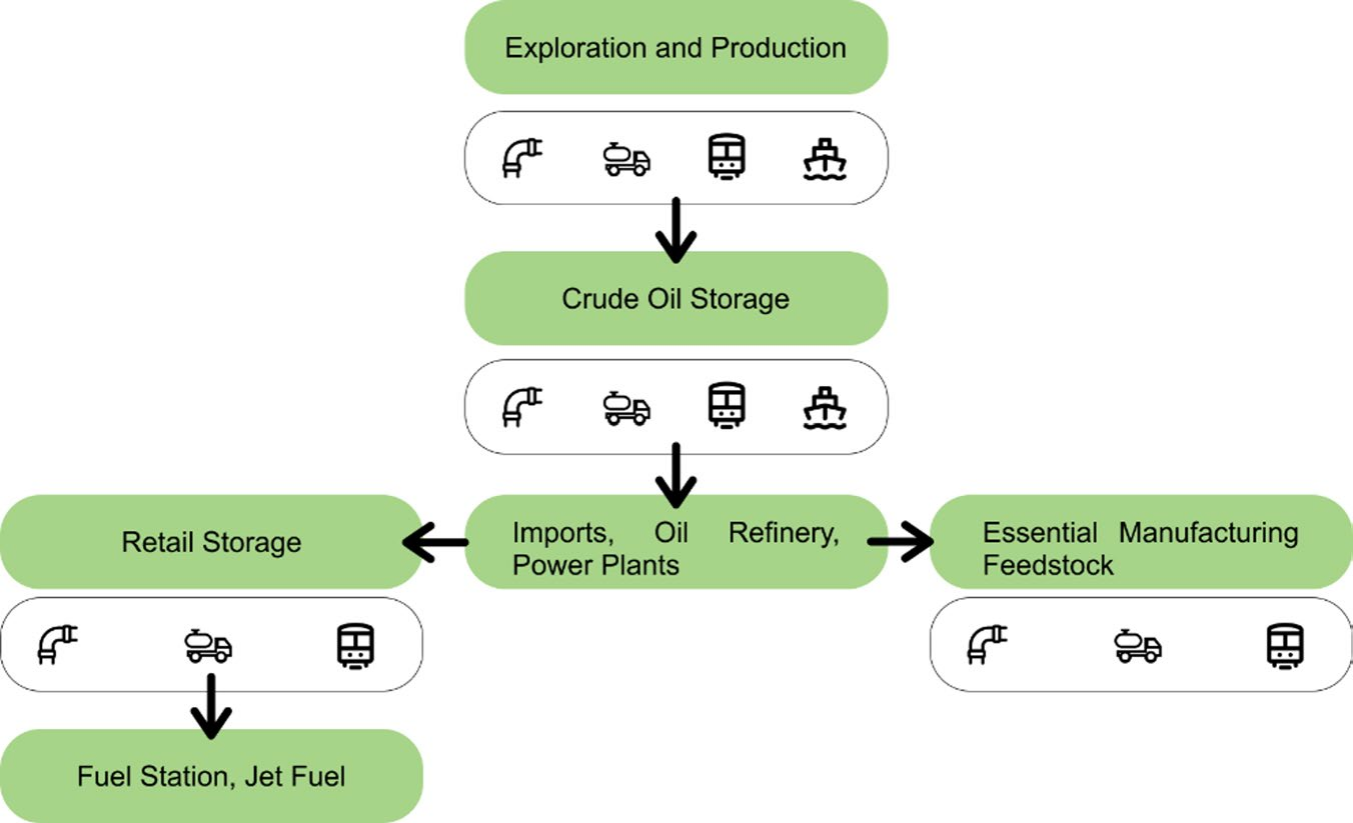
Simplified Downstream Oil Supply Chain.

The primary focus of this study is the development of an optimization model for the downstream oil supply chain (DOSC), emphasizing a multi-echelon, multi-product, and multi-modal transport network design. DOSC involves all the operations that begin after the production phase continue till the sale to the end user. The processes involve crude oil refining, petroleum product distribution, storage of refined fuels, and transportation to retail outlets. This model aims to determine the minimum cost required to meet overall refined product demands, assessing performance under various disruption scenarios. Through the application of a MILP model and a Monte Carlo sampling technique, the study presents a real-world application, showcasing the influence of uncertainties on the supply chain’s dynamics and cost structure. While uncertainties lead to increased costs, they drive adaptive changes, enhancing the supply chain’s resilience without significant escalation in transportation costs. The secondary objective of this research is to observe the effects on the flows between entities in the POSC. The disruptions in supply and demand sides will result in the change of flow between entities and hence the overall supply network cost. These disruptions can be random or anticipated depending on the scenario. In each scenario, the minimum cost would be calculated for the overall demand. The goal will be to analyze the effect on cost with the variation in parameters. Different disruptions would have separate impacts on the supply chain, resulting in variation of the total network cost. This would also be helpful in creating mitigation strategies for possible disruption scenarios. This study addresses a key research gap by examining an import dependent country’s downstream oil network under realistic infrastructure and capacity constraints. Much of the existing literature either focuses on oil-rich countries with abundant reserves or assumes extreme scenarios with complete supply chain shutdown. Prior studies employ models that emphasize profit maximization or propose hypothetical network infrastructure expansions; this study prioritizes transport cost minimization within the existing network infrastructure and aims to provide a more practical decision support framework. Furthermore, the use of GIS based distance mapping with MILP optimization, and the application of Monte Carlo simulation offers a novel methodological contribution, which enables a more accurate and realistic representation of the DOSC under uncertainty. A simplified scheme of a downstream oil supply chain is shown in Fig. 1.

## Literature review

### Modeling approaches in petroleum supply chain

Supply chain management in the petroleum industry is filled with various problems and challenges, especially pertaining to logistics and inventory, which are not common in most other process industries. Hussain et al.^6^, have shed some light on the challenges and opportunities in the petroleum industry and practices which are used by industry giants around the world. Lima et al.^7^,reviewed the utilization of mathematics-based programming techniques in case of distribution-oriented problems, particularly by the multiple entities involved in the downstream oil supply chain. With the increase in complexity, the number of variables involved and the difficulty in coordinating between them also increase. Sinha et al.^8^, implemented multi-agent technology to make the supply chain faster compared to conventional supply chain practices. The complexity in a PSC leads to the creation of various forms of risks that need to be understood when planning, designing and operating such an environment. Amor et al.^4^, present a detailed review on the risks associated with the oil and gas industry and provide an understanding to further develop risk management techniques. The study classified key risks related to every segment of the PSC. The results expressed that the type of risk is dependent on certain operations of the PSC and whether the country was an exporter or an importer. While prior studies offer valuable modelling frameworks, they often treat risks and disruptions separately. This study builds on this foundation and compounds disruption effects on the downstream network.

### Supply chain design and optimization

Designing and optimizing downstream oil supply chains has been a core research focus. Fernandes et al.^9^, takes into account a DOSC network with common resource capacities, installations, demand requirements, and supply sources that includes several entities, echelons, multiple products, and multiple modes of transportation. The MILP is evaluated using an actual Portuguese PSC network, which involved local crude oil processing refineries for production and a local hub for supply. Wang et al.^10^, created a MILP model that optimizes a DOSC by developing new pipeline routes. Focusing on the distribution of refined oil products between storage facilities and using real-world data, the model obtained new routes for pipelines. A case of a DOSC in China was conducted with multiple situations analyzed. A thorough analysis of the efforts put forth in optimizing natural gas transmission lines was given by Mercado et al.^11^. It addressed both steady-state and transient optimization models, emphasizing the modelling features and current best practices for solution methods. These works optimize structural aspects such as routes, depots and capacities; however, they mostly assume stable operating conditions. This study extends their insight by considering dynamic disruption scenarios within the designed model.

### Uncertainty in petroleum supply chain

Uncertainty is inherent in oil supply chains due to demand and supply fluctuation, price volatility, and operational disruptions. One of strategies to overcome uncertainty in energy supply chains is to strategically and tactically plan DOSC with subject to various uncertainty sources. Lima et al.^12^, formulated a mixed-integer linear programming model (MILP) in which uncertainty was accounted for, by using chance constrained programming with fuzzy parameters. The network was designed by determining the strategic locations to hold warehouses, while their capacities are established by installing specific tanks for each product. Al-Othman et al.^13^, developed a planning model for a multi-stochastic petroleum supply chain network and implemented it under uncertain market scenarios for an oil producing country. Uncertainties were in the form of demand and price of oil products. Scheduling refinery maintenance to prevent unexpected shutdowns is one of the issues faced by the petroleum sector. Refineries, hubs, depots, and customers make up the four tiers of the petroleum industry’s multi-echelon supply chain network according to Khosrojerdi et al.^14^,. It demonstrated how the optimum objective value and solution can be significantly impacted by the uncertainty in parameters like the quantity of refineries that are accessible, price, and demand. While these models handle single uncertainty dimensions (demand, price, or maintenance), the current study explores simultaneous disruptions to capture their interactive effects on their flow of petroleum products.

### Disruptions in petroleum supply chain

Climate-induced events can pose serious challenges to PSCs, mostly in terms of facility breakdowns. Jabbarzadeh et al.^15^, investigated a supply chain design issue with the potential for facility disruptions. The facilities are always susceptible to various forms of disruptions brought on by calamities, intentional defects, and equipment failures. They formulated the issue as a mixed-integer nonlinear program that seeks to maximize the overall system profit. Climate change has intensified and increased the frequency of extreme weather events which has resulted in uncertainties in demand, production, and inventory in supply chains. Ni et al.^16^, proposed a systematic methodology to evaluate the flexibility of supply chains and applied it to demand side management of a DOSC. Several scenarios of disruption in refinery operations, pipeline shutdowns, and increase in demand were generated. The fluctuating demand and prices of oil products forces companies to redesign their existing distribution networks and production strategies to improve overall network flexibility and reduce costs. A stochastic mixed integer linear problem is presented for the PSC design by Fernandes et al.^17^,. The objective was to design the network under demand uncertainty that maximizes expected net present value (ENPV) of a multi-entity multi-product PSC network. The decisions variables involve depot capacities, transport modes, locations of storage facilities and inventory. The studies analyzed models with single event disruptions. The novelty of this research lies with analyzing multiple simultaneous disruptions which reflect real world uncertainty complexities.

### Strategic and tactical planning

PSC design requires coordination across strategic (facility placement and capacity investment) and tactical levels (flow allocation and scheduling). Zhao et al.^18^, examined China’s energy security from a supply chain viewpoint with regards to economic and geo-political issues. They concluded that there is an inherent threat due to the interconnectedness of China’s oil system. For optimum long-term scheduling and planning of a refined oil products supply chain, a three successive stage (echelon) model was developed by Alfares^19^. The stages are divided into refineries and import terminals, distribution centers, and demand markets. The strategic element involves the placement and construction of refineries and distribution centers while the tactical element involves the flow of products between entities. The creation of a strategic planning model for an integrated oil chain is suggested by Ribas et al.^20^,, considering these sources of uncertainty: refined product demand, crude oil output, and market prices. The findings reveal large income disparities between the formulations, depending on the agent’s risk profile. A deterministic MILP model was proposed by Kazemi et al.^20^, that determined the optimal distribution center locations, capacities, transfer volumes and transportation modes. The model, implemented on US downstream oil supply chain, minimized the multi echelon, multi-product cost along the refineries, depots, transport modes and fuel station (demand nodes). It also proposed proactive mitigation strategies in the form of varied transfer volumes and for reactive mitigation strategies it employed multimodal transportation strategies. In a distribution network that includes refineries, ports and few pipeline systems, the study by Más et al.^21^ handles short-term crude oil scheduling issues. First, a MILP model is suggested that takes into account a combined depiction of the pipeline and intermediate storage facilities. The assignment of tank lorries to oil piers, as well as vehicle filling and pipeline freight procedures, are decision factors. Multi-product pipelines often distribute multiple goods to designated delivery points in batches. In order to improve the detailed scheduling of a multi-product pipeline with many pump stations, Liao et al.^22^ created a continuous-time mixed-integer linear programming (MILP) model based on flowrate database. Bastidas et al.^23^ produced a thorough analysis of the supply chain models’ freight transportation function, and various transportation-related features were noted. Regarding transportation modelling, they discovered one paradigm, two trends, and an outlier. By taking into account demand, supply costs, and selling prices, Escudero et al.^24^ created a two-stage model for supply and distribution scheduling of a multi operator multi product petroleum supply chain. A broad framework for simulating petroleum supply chains is the main goal of Neiro et al.^25^. The chain’s nodes are viewed as a collection of elementary elements connected by intermediary streams. The nodes that represent the pipeline networks, refineries, and terminals are then connected to create complicated topology. Stream flow rates, attributes, operational factors, inventory, and facility assignment are decision variables. These studies demonstrate the value of integrated planning with multiple dimensions such as transportation routes, flow allocations and multiple operators. This study evaluates the cost function and variation in flows in downstream network disruption scenarios.

### Solution techniques

Heuristics and linear programming were both used in early applications. Numerous formulations and approaches to solving problems have been employed, such as continuous and discrete optimization, stochastic programming to deal with uncertainty, and metaheuristics like genetic algorithms to improve solution quality while minimizing computational load. The prevalence of using numerical optimization or mathematical programming techniques to build and operate petroleum fields, allocate lift gas and rate, and construct, plan, and manage reservoirs was examined by Khor et al.^26^. Sangaiah et al.^27^ discuss a solid mixed-integer linear programming model for planning Liquified Natural Gas sales over a specified time horizon with the goal of reducing vendor costs. A novel hybrid solution to the p-hub center problem is presented by Bashiri et al.^28^, in which the hub facilities’ location is simultaneously dictated by two factors. Fuzzy systems are employed as the foundation of this work since they are used to handle qualitative and ambiguous data. The paper models a hybrid hub solution using fuzzy VIKOR. Samadi et al.^29^ take into account two factors: cost and environmental impact. The Sri Lankan petroleum supply chain was considered to analyze risk factors and disruptions. An expert opinion survey was conducted coupled with a probability impact analysis to assess risk factors. The findings by Fernando et al.^30^,highlighted that a lack of infrastructure facilities is the main hurdle that stops the country from ensuring energy security. Therefore, a forecasting model was developed utilizing ARIMA to determine future demand for the product. The results illustrate that in the next two years; additional shipments will be required on a monthly basis. Many advanced techniques have been utilized to provide complex solutions but are not able to practical adaptability and interpretability. By combing MILP with Monte Carlo simulation, this research provides a reasonable applicable framework for real world applications.

### Research gap

The research gap involves finding the effects of multiple disruptions occurring simultaneously and individually on a supply chain. There are multiple factors that are involved in the mechanisms of an operating supply chain. Therefore, it is necessary to understand the impacts of these factors on both micro and macro levels. Focusing on the reliable design of refined oil product supply chains, which are essential to industry performance, a MILP model coupled with a Monte Carlo sampling of parameters like demand uncertainty, oil products and hub disruptions were generated. A real-world case is used to understand the accuracy, efficiency and applicability of the proposed model. The results would provide an understanding of the effects of uncertainty on the supply chain. The total cost involving the uncertainties is relatively higher than without uncertainties. Uncertainties are also responsible for the change in final construction scheme of the supply chain. However, design changes under uncertainties can improve the reliability of the supply chain without much increase in the overall transport cost.

Although the proposed model follows the general framework of capacity-constrained resource allocation problem, it introduces several novel features tailored to DOSC. Firstly, the formulation integrates a multi-echelon, multi-product, and multi-modal network simultaneously whereas most prior studies only address only single layers or transport modes in isolation. Secondly, the model explicitly incorporates Monte-Carlo based disruption scenarios to study effects simultaneous supply and demand side uncertainties on flows, costs, and resilience. This contrasts with conventional deterministic or single disruption models. Thirdly, unlike profit maximization models requiring extensive operational and asset data, the present study emphasizes transport cost minimization, which reflects the dominant driver of downstream logistics efficiency in petroleum distribution. Finally, the model is validated on a real-world nationwide case, demonstrating how disruptions alter cost structures and flow allocations across pipelines, rail, and road networks. Together, these elements distinguish the proposed model from traditional capacity-constrained formulations and provide a practical decision-support tool for industry and policymakers.

## Model development

### Proposed approach

The aim of developing the model is to simulate a refined petroleum products supply chain, comprehending the intricate dynamics of a multi-modal and multi-product network configuration and performance evaluation under diverse conditions. The proposed model serves as a representation of the designed supply chain network, utilizing parameters based on the average values obtained from the collected data. It serves as a fundamental framework for analyzing the intricate flows between entities and the inventory management at storage facilities. Employing Geographic Information System (GIS), the model utilizes the geographical coordinates of storage facilities and refineries to accurately estimate transport costs, particularly for road transportation networks. The initial deterministic model uses parameters in an environment having no disruptions, which will provide baseline values for material flow and total network costs. Later, the results from the initial model would be compared to models having disruptions in their supply chains.

Furthermore, another model is devised to account for disruptions in the supply chain, considering the impact of both demand and supply issues. This model optimizes the flow between entities, adapting to variations in the parameters associated with disruptions, which can be categorized as either random or predetermined. Based on the model’s results, potential mitigation strategies can be recommended to alleviate potential hindrances that might impede the smooth operation of the entire supply chain.

In the proposed MILP model, it is assumed that there exists a set R of refineries denoted by r, with respective supply capacities (Sr), a set D of distribution centers (depots) represented by d with product-specific capacities (Ud), and a set FS of demand nodes (fuel stations) designated as fs, with corresponding product demands denoted by p. The transport mode set T includes three modes (road, rail, and pipe). The complete notation utilized in the proposed model is delineated in Table 1.

**Table 1:** Description of Indices, Parameters, and decision Variables.

Category	Description
**Indices**	
*R*	Index of refineries; *r* ε *R*
**D**	**Index of depots; d** ε **D**
**I**	**Index of ports; I** ε **I**
**Fs**	**Index of fuel stations; f s** ε **FS**
**T**	**Index of transport modes; t ε T**
**P**	**Index of products; p ε P**
	**Parameters**
**D f s**,** p**	**Daily demand for fuel stations for product p**
**C r**,** d**,**p**,** t**	**Cost of transport per unit from refinery r to depot d for product p via transport mode t**
**C i**,** d**,**p**,** t**	**Cost of transport per unit from port i to depot d for product p via trans- port mode t**
**C d1**,**d2**,**p**,** t**	**Cost of transshipment per unit from depot d1 to depot d2 for product p** **via transport mode t; d1** /**= d2**
**C d**,** f s**,** p**,**t**	**Cost of transport per unit from depot d to fuel station fs for product p** **via transport mode r**
**S r**,** p**	**Daily supply capacity of refinery r for product p**
**S i**,** p**	**Daily supply capacity of port i for product p**
**U d**,** p**	**Storage capacity of depot d for product p**
	**Decision Variables**
**V r**,** d**,**p**,** t**	**Volume of product p transported from refinery r to depot d via transport** **mode t**
**V i**,** d**,**p**,** t**	**Volume of product p transported from port i to depot d via transport mode t**
**V d1**,**d2**,**p**,** t**	**Volume of product p transported from depot d1 to depot d2 via transport mode t; d1** /= **d2**
**V d**,** f s**,** p**,**t**	**Volume of product p transported from depot d to fuel station fs via transport mode t**
**Category**	**Description**
**Indices**	
*R* *D I* *Fs* *T P*	Index of refineries; *r* ε *R* Index of depots; *d* ε *D* Index of ports; *I* ε *I* Index of fuel stations; *f s* ε *FS* Index of transport modes; *t* ε *T* Index of products; *p* ε *P*
**Parameters**	
*Df s*,*p* *Cr*,*d*,*p*,*t*	Daily demand of fuel stations for product *p* Cost of transport per unit from refinery *r* to depot *d* for product *p* via transport mode *t* Cost of transport per unit from port *i* to depot *d* for product *p* via trans- port mode *t* Cost of transshipment per unit from depot *d*1 to depot *d*2 for product *p* via transport mode *t*; *d*1 /= *d*2 Cost of transport per unit from depot *d* to fuel station *f s* for product *p* via transport mode *r* Daily supply capacity of refinery *r* for product *p* Daily supply capacity of port *i* for product *p* Storage capacity of depot *d* for product *p*
*Ci*,*d*,*p*,*t*	
*Cd*1,*d*2,*p*,*t*	
*Cd*, *f s*,*p*,*t*	
*Sr*,*p* *Si*,*p* *Ud*,*p*	
**Decision Variables**	
*Vr*,*d*,*p*,*t*	Volume of product *p* transported from refinery *r* to depot *d* via transport mode *t* Volume of product *p* transported from port *i* to depot *d* via transport mode *t* Volume of product *p* transported from depot *d*1 to depot *d*2 via transport mode *t*; *d*1 /= *d*2 Volume of product *p* transported from depot *d* to fuel station *f s* via transport mode *t*
*Vi*,*d*,*p*,*t*	
*Vd*1,*d*2,*p*,*t*	
*Vd*, *f s*,*p*,*t*	

The supply chain proposed model includes the location of fuel storage facilities, refineries, and fuel stations along with their capacities. It also includes three modes of transport: flow of products from refineries to depots, transshipment of products between depots, and transport of products from depots to fuel stations. The model has the ability to choose from multiple modes of transportation to move products, provided if a certain mode is available. The following decisions need to be optimized considering the constraints of each entity in the supply chain:

- Volume of products transported from refinery to depots.
- Volume of products transported from ports to depots.
- Volume of products transshipped between depots.
- Volume of products transported from depots to fuel stations.

The objective function (1) minimizes the overall transport cost of the downstream oil supply chain from the refineries to the depots and from the depots to the fuel stations. The first term represents the combined cost of transport of products from refineries to depots, the second term represents the cost of transport of products from ports to depots while the third term is the transshipment cost between depots. The last term accounts for the cost to transport products from depots to fuel stations. Constraint (2) ensures that the demand for fuel at each fuel station is satisfied by the distribution centers. Constraint (3) limits the supply from the refineries to depot up to the refinery production limit. Constraint (4) ensures that the inflow of products from refineries, ports and other depots does not go beyond the storage capacity of products at each storage facility. Constraint (5) is the conservation of material at the storage facilities so that the flow of products to fuel station is not more than the flow of products into depots. Constraints (6) are non-negativity of the decision variables.

The mathematical formulation of the model is presented below.

Minimize:

1 $$\begin{gathered} \sum\limits_{{r\epsilon R}} {\sum\limits_{{d\epsilon D}} {\sum\limits_{{p\epsilon P}} {\sum\limits_{{t\epsilon T}} {{V_{r,d,p,t}} \cdot {C_{r,d,p,t}}} } } } \hfill \\ +\sum\limits_{{i\epsilon D}} {\sum\limits_{{d\epsilon D}} {\sum\limits_{{p\epsilon P}} {\sum\limits_{{t\epsilon T}} {{V_{i,d,p,t}} \cdot {C_{i,d,p,t}}} } } } \hfill \\ \quad +\sum\limits_{{d1\epsilon I}} {\sum\limits_{{d2\epsilon D}} {\sum\limits_{{p\epsilon P}} {\sum\limits_{{t\epsilon T}} {{V_{d1,d2,p,t}} \cdot {C_{d1,d2,p,t}}} } } } \hfill \\ \quad +\sum\limits_{{d\epsilon D}} {\sum\limits_{{fs\epsilon FS}} {\sum\limits_{{p\epsilon P}} {\sum\limits_{{t\epsilon T}} {{V_{d,fs,p,t}} \cdot {C_{fs,d,p,t}}} } } } \hfill \\ \end{gathered}$$

Subject to:

2 $$\sum\limits_{{d \in d}} {\sum\limits_{{fs \in FS}} {\sum\limits_{{p \in P}} {\sum\limits_{{t \in T}} {{V_{d,fs,p,t}} \cdot {D_{fs,p,}}} } } }$$

3 $$\sum\limits_{{r \in R}} {\sum\limits_{{d \in D}} {\sum\limits_{{p \in P}} {\sum\limits_{{t \in T}} {{V_{r,d,p,t}} \leqslant {S_{r,p}}\forall {\kern 1pt} r\epsilon R,p\epsilon P} } } }$$

4 $$\sum\limits_{{i \in I}} {\sum\limits_{{d \in D}} {\sum\limits_{{p \in P}} {\sum\limits_{{t \in T}} {{V_{i,d,p,t}}} } } } \leqslant {S_{i,p}}\forall {\kern 1pt} r\epsilon R,p\epsilon P$$

5 $$\begin{gathered} \sum\limits_{{r\epsilon R}} {\sum\limits_{{d\epsilon D}} {\sum\limits_{{p\epsilon P}} {\sum\limits_{{t\epsilon T}} {{V_{r,d,p,t}}+\sum\limits_{{i\epsilon I}} {\sum\limits_{{d\epsilon D}} {\sum\limits_{{p\epsilon P}} {\sum\limits_{{t\epsilon T}} {{V_{i,d,p,t}}} } } } } } } } \hfill \\ +\sum\limits_{{d1\epsilon D}} {\sum\limits_{{d2\epsilon D}} {\sum\limits_{{p\epsilon P}} {\sum\limits_{{t\epsilon T}} {{V_{d1,d2,p,t}} \leqslant {\kern 1pt} {\kern 1pt} {U_{d,p}}\forall d\epsilon D,p\epsilon P} } } } \hfill \\ \end{gathered}$$

6 $$\begin{gathered} \sum\limits_{{r\epsilon R}} {\sum\limits_{{d\epsilon D}} {\sum\limits_{{p\epsilon P}} {\sum\limits_{{t\epsilon T}} {{V_{r,d,p,t}}+\sum\limits_{{i\epsilon I}} {\sum\limits_{{d\epsilon D}} {\sum\limits_{{p\epsilon P}} {\sum\limits_{{t\epsilon T}} {{V_{i,d,p,t}}} } } } } } } } \hfill \\ +\sum\limits_{{d1\epsilon D}} {\sum\limits_{{d2\epsilon D}} {\sum\limits_{{p\epsilon P}} {\sum\limits_{{t\epsilon T}} {{V_{d1,d2,p,t}}=\sum\limits_{{d\epsilon D}} {\sum\limits_{{fs\epsilon FS}} {\sum\limits_{{p\epsilon P}} {\sum\limits_{{t\epsilon T}} {{V_{d,fs,p,t}}} } } } } } } } \hfill \\ \end{gathered}$$

Variables.

$$\begin{gathered} {V_{r,d,p,t}},\,{V_{i,d,p,t}},\,{V_{d1,d2,p,t}},\,{V_{d,fs,p,t}} \geqslant 0 \hfill \\ \forall r\epsilon R,\,i\epsilon I ,\,d,d1,d2\epsilon D,fs\epsilon FS,p\epsilon P,t\epsilon T \hfill \\ \end{gathered}$$

The deterministic model serves as the foundation for the subsequent Monte Carlo simulations. In the Monte Carlo simulation, the parameters of the transport cost per unit for each mode are adjusted, in addition to the supply of products from refineries and the demand of products at fuel stations. The network structure remains consistent during the Monte Carlo simulations, mirroring the deterministic case. The capacities of depots and the distances between all entities remain unchanged in both scenarios.

### MONTE CARLO simulation based on the proposed model

The Monte Carlo simulation is a technique used for conducting sensitivity analysis, aimed at understanding how a model reacts to inputs generated in a stochastic manner. Typically, this process involves the random generation of inputs, or scenarios, followed by the execution of a simulation for each of these scenarios using a computerized model. The resulting outputs from these scenarios are evaluated and aggregated. Statistical analysis of these results provides key insights, including the mean value, output distribution, and the operational range of the model. Varying parameter values, encompassing fuel station demands, refinery supplies, and transport costs between entities, require the generation of distinct scenarios through Monte Carlo sampling. Each of these uncertain optimization models is then transformed into a deterministic optimization problem in its own right. The Python 3.11 programming language is employed to build the designed model, with PuLP 2.7 serving as the primary solver. To comprehensively explore the impacts of uncertainties on the model, several general cases for the scenarios are generated, including:

- Disruption 1: High demand and low refinery output.
- Disruption 2: Low demand and constant refinery output.
- Disruption 3: Escalating transport costs per unit of fuel due to the cost-of-living crisis.
- Disruption 4: The breakdown of the pipeline network, necessitating repair and leading to a shift in the transportation of oil.

The Monte Carlo simulation employs all these scenarios simultaneously to generate the flows of products between entities. The analysis involves assessing the simultaneous alterations to multiple parameters within the supply chain. Even though stochastic optimization and scenario analysis are widely used for uncertainty modelling, they face limitations in DOSCs. Stochastic optimization requires precise probability distributions, which are rarely available for disruptions such as refinery shutdowns, transport interruptions, or sudden demand shifts. Scenario analysis, on the other hand, evaluates only a limited number of predefined cases and cannot capture the full variability of real-world conditions. Monte Carlo simulation was therefore chosen for its ability to systematically sample from estimated ranges of uncertain parameters and generate a large number of possible realizations. By repeatedly solving the MILP model under these realizations, the approach provides not only expected costs but also the variance and risk profile of the supply chain. This makes Monte Carlo particularly valuable for assessing resilience, as it quantifies how disruptions of different magnitudes and frequencies influence flows and costs across the network.

### Parameter setup

To assess the efficacy of the formulated model, a practical case study of a downstream petroleum supply chain for a company is selected. The country’s population distribution indicates that the highest demand for transportation fuel lies in the northeastern province of Punjab. Sindh, particularly Karachi, serves as the nation’s most significant industrial center and accounts for the second-highest fuel consumption. On the other hand, the demand for fuel is relatively low in KPK and Baluchistan due to limited industrialization and a scattered population. However, since the majority of refineries and import terminals are situated in the southern part of the country, transportation of fuel to the north is necessary to meet the demand.

For the initial deterministic model, the parameters are configured based on the company’s daily refined fuel sales nationwide, alongside the average daily refinery output. The refineries production capacities vary between 0.8 million to 3.0 million liters per day; however, an average output is utilized for the initial model. The storage capacity of depots also varies, with the largest being close to 160 million liters while the smallest is around 0.2 million liters. The pipeline and railway networks are obtained from government reports, while the road network is established using GIS data. In this deterministic model, 3,500 fuel stations are considered, representing approximately 36% of the total operational fuel stations in the country, without any provincial divisions due to inter-provincial sale instances. The country is equipped with 5 refineries, each contributing to the supply of products according to their capacities. Additionally, 25 storage facilities are distributed across the nation, each with specific product capacities based on actual distribution requirements. No disruptions or capacity constraints are incorporated in this model, allowing for the verification of a fully operational model with all associated constraints.

The MILP model is designed to ascertain the minimized cost of transporting refined fuel products in accordance with the demands of fuel stations. The primary decision variables include the flow of products between entities via various transportation modes. Modeling for random disruptions involves the alteration of parameters through scenario generation, adhering to standardized deviation criteria.

## Solution, procedure and results

The comparison would involve assessing the flow of products between various entities and the total cost of the supply chain network in the deterministic model versus the distinct case scenarios. Furthermore, the results obtained will offer valuable insights into the supply chain’s performance when disruptions lead to variations in its parameters. As the cases can be distinguished between 1 and 2, potential mitigation strategies can be recommended to minimize the impact of disruptions on the supply chain’s operations. All models are coded in Python, with the PuLP solver utilized to achieve model optimality while minimizing the overall transport cost.

### Computational results for the deterministic model

This model comprised 179,600 variables and 7,110 constraints, which were efficiently solved in 2.36 s with 7,793 iterations, yielding an overall network cost of 1,067,342,436 PKR. To enhance the cost-effectiveness of supplying the northern region, only those depots with additional pipeline connections to other depots were equipped with pipeline fuel supply. With an excess of HSD production by the domestic refineries, no further imports were necessary through the ports. Nevertheless, to compensate for the lower PMG supply, significant volumes were allocated to the depots connected to the terminals via pipelines. The least utilized mode of transport from the refinery to depots was through roads. For the mid-country refinery, rail transport was employed for both fuels to facilitate transportation in the central region, considering not all depots were connected by pipelines. While there was minimal transshipment between depots for HSD, the demand at the fuel stations was directly fulfilled from depots via road transport. However, some volume of HSD was transported through pipelines. Given the higher demand for PMG, a relatively larger proportion of volumes were transshipped using both roads and pipelines. The comprehensive transportation structure is presented in Table 2, reflecting that most of the fuel movement occurred via roads, encompassing the final stage of the supply chain, namely, shipments to fuel stations. Pipelines were the second most utilized mode of transport, ensuring efficient and cost-effective movement to the up-country regions. Considering the limited railway network in the country, only a fraction of the transportation was facilitated through rail carriages.

**Table 2:** Fuel movement by transport mode in the deterministic model.

Product	Road	Rail
HSD	12,278,723	2,539,538
PMG	17,408,147	3,055,137

The volume of flows of refined products from the refineries is detailed in Table 3. As anticipated, the refineries are primarily supplying a greater volume of PMG compared to HSD, reflecting its prevalent use in the transportation industry. Notably, the northern refineries contribute significantly to the supplies, leveraging their proximity to the replenishing storage facilities for meeting demand nodes. In the southern region, all three refineries primarily cater to the energy and transport needs while also channeling their products northward. Individually, the volume of road transport is minimal from the refineries. Wherever rail tracks are accessible, the refineries prefer the use of train wagons, in addition to pipelines. Opting for the pipeline mode of transportation is preferred across all refineries, considering its cost-effectiveness and safety in fuel transportation to the storage facilities.

**Table 3:** Transportation volumes (in tons).

Refineries	Depots					
	Road (HSD)	Road (PMG)	Rail (HSD)	Rail (PMG)	Pipe (HSD)	Pipe (PMG)
1	12,588	-	184,596	-	1,818,167	2,200,041
2	121,439	1,269,769	184,510	1,460,323	1,975,554	1,829,176
3	36,194	-	176,767	-	2,027,968	817,845
4	54,204	-	185,121	888,038	1,004,720	566,933
5	102,427	-	249,986	-	1,760,221	1,303,562
**Total**	326,852	1,269,769	980,980	2,348,360	8,586,630	6,717,556

The output of the ports, detailed in Table 4, demonstrates a preference for PMG imports over HSD, in line with the extensive use of PMG across the country. Port 1, in particular, solely imports PMG and refrains from importing any HSD. Given the existence of the white oil pipeline, connected with storage facilities nearest to these ports, it is logical to utilize these ports for fulfilling the demands in the upcountry regions.

**Table 4:** Transportation volumes (in tons) –- ports to depots (Pipe).

Ports	Depots (Pipe)	
	HSD	PMG
1	-	1,705,883
2	1,004,719	1,303,561

Table 5 reflects the subsequent stage of the supply chain, outlining the transshipment of fuel between depots. Predominantly, the transshipment occurs via pipelines, with PMG accounting for the larger share. As the railway network between storage facilities remains non-operational, there is no fuel movement facilitated by train wagons. However, certain peripheral facilities lacking pipelines or train tracks rely on road transport to supply both PMG and HSD. During the final phase of this supply chain, all movement is orchestrated through fuel carriages via road transport, corresponding to the daily demand of fuel stations nationwide. The higher volume of PMG is indicative of its extensive use in the transportation sector, while HSD finds more application in heavy vehicles and construction equipment.

### Computational results for the case wise Monte Carlo simulations

Monte Carlo simulations are implemented to generate various scenarios based on the deterministic model. The simulation employs statistical means with distinct standard deviations to produce diverse parameters for the mode’s operation. Normal Distribution is used to generate scenarios by modelling uncertainty in terms of cost, demand and production. The standard deviation lies between 0 and 1 to avoid having irregular parameters in the model. While the parameters are generated individually, the final simulation incorporates all cases to emulate real-world scenarios.

#### Disruption 1

**Fig. 2:**
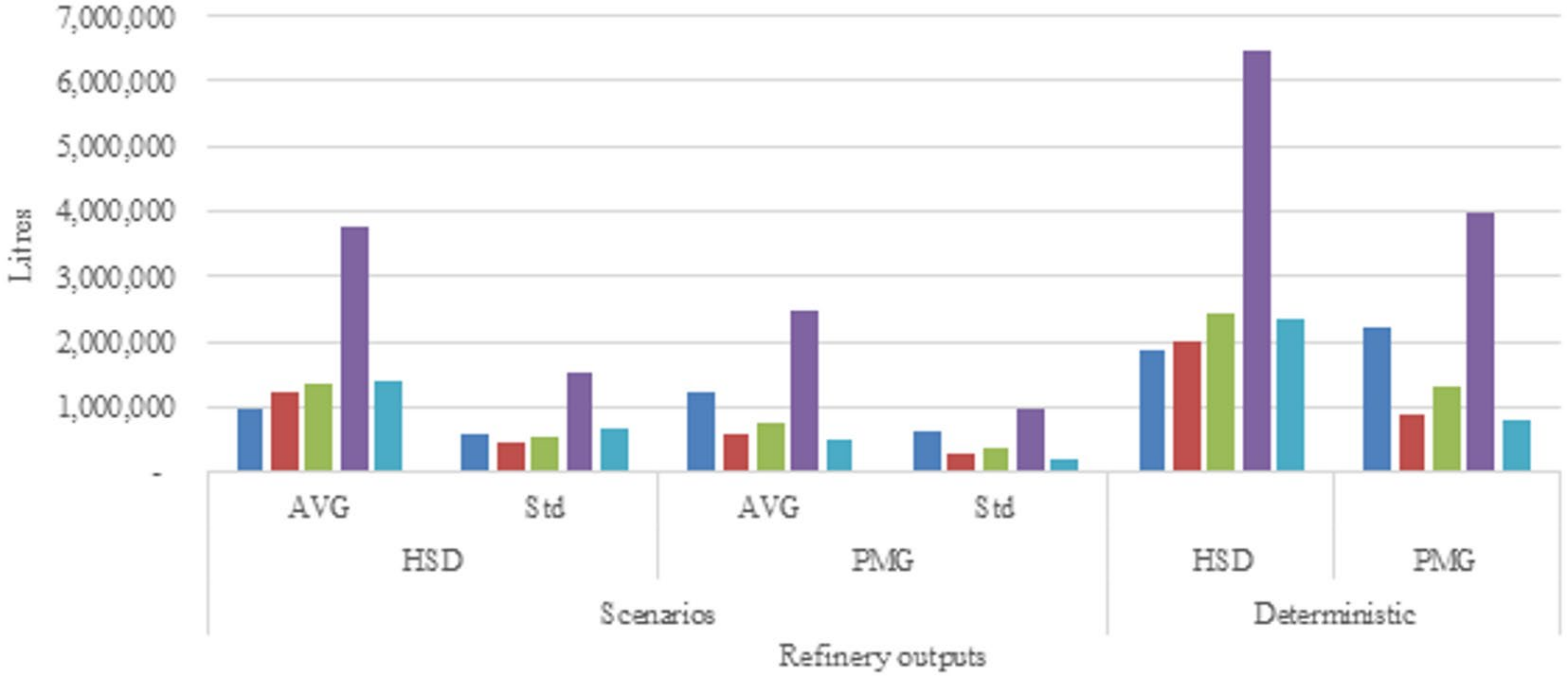
Scenario-wise refinery output.

A decrease in the supply of products from refineries prompts an imbalance in the supply chain. Notably, there is an overall reduction in production capacity for both HSD and PMG by approximately 42% and 40%, respectively. Several factors could contribute to this decline, including challenges in crude oil sourcing, reduced operations due to inadequate storage capacity for less in-demand products, or pricing disputes between refineries and regulators. Multiple scenarios with reduced refinery production capacity are generated based on these values, and the average is computed for various reduced-capacity scenarios. Figure 2 illustrates the scenarios generated for Disruption 1.

#### Disruption 2

The demand from fuel stations has dramatically surged compared to the fuel station demand outlined in the deterministic model. The post-COVID-19 recovery in economies has triggered a substantial escalation in the need for refined petroleum products. This surge can be attributed to the increased demand for transportation, energy requirements, and industrial complexes ramping up their production, thereby intensifying the consumption of petroleum-based products as their raw materials. The demand increase is documented in Table 6, demonstrating a 22% surge in overall demand for HSD and a 32% increase for PMG.

**Table 5:** Transshipment volumes (in tons) between Depots.

Depots	Transshipment Volumes (Road, Rail, Pipe)					
	Road (HSD)	Road (PMG)	Rail (HSD)	Rail (PMG)	Pipe (HSD)	Pipe (PMG)
HSD PMG	8,498 -	- -	75,626 -	- -	1,126,305 -	4,119,280 -

**Table 6:** Scenario averages (in Tons).

Scenario	HSD (Average)	PMG (Average)	HSD (Deterministic)	PMG (Deterministic)
1	14,118,338	21,770,312	11,509,257	16,410,111

#### Disruption 3

The per-unit cost of transporting refined fuel products has escalated due to the surging cost of fuel. The burgeoning demand for refined fuel, coupled with inadequate supplies from refineries, has caused a shortage, leading to an increase in the per-unit cost. Notably, the transportation of fuel from one point to another necessitates a significant amount of energy. Vehicles transporting fuel between entities, pumps facilitating the flow of fuel within pipelines, and train engines hauling oil wagons all consume fuel during operations. The elevated procurement cost of fuel has consequently led to an increase in transportation costs, divided on a per-unit basis for transported products. This is particularly crucial as all stages require energy for the movement of oil products. Notably, the most significant change in transport cost per unit of fuel was observed in movement through pipes, followed by movement by road, with the least change recorded in movement via rail. The minimal change in the rail category is attributed to the limited sample size used to generate the scenarios.

#### Disruption 4

The primary pipeline, serving as the most cost-effective mode of transportation between the southern refineries and the northern markets, as outlined in the deterministic model, is disrupted. In the event of such a disruption, the burden on road and train transportation would substantially increase. Pipelines function as the backbone of the downstream oil supply chain, facilitating the direct pumping of imported refined oil products from terminals to storage facilities in the central regions of the country. Any disruption in the operations of the WOP (white oil pipeline) would have catastrophic repercussions for the entire supply network. Refineries would be compelled to maximize fuel production, necessitating the road and railway networks to shoulder the load previously handled by the pipeline.

The Monte Carlo simulation is utilized to analyze the flow of refined product volumes between entities across the supply chain network for all the aforementioned scenarios. Given that changes in the supply parameters can lead to variations in the optimal solution, the objective of this simulation is to incorporate multiple scenarios for all cases and determine an optimal solution. The multi-scenario model accounts for uncertainties stemming from both external and internal factors. Comparisons with the deterministic model will be used to refine the supply chain parameters, including depot capacity, transport mode constraints, and depot locations. A total of 50 scenarios are generated for each disruption, and the same constraints are applied as in the deterministic model.

## Sensitivity analysis

### Impact of uncertainty in supply chain parameters on the supply network cost

**Fig. 3:**
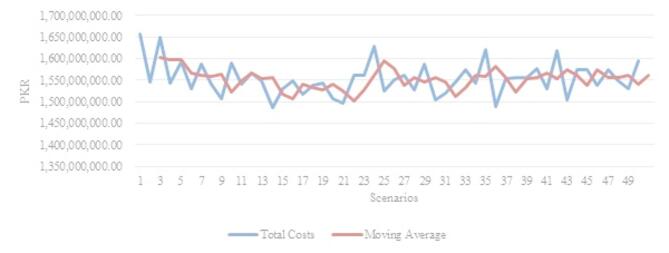
Scenario-wise Minimized Network Transport Cost (PKR).

As anticipated, the overall network cost has experienced a substantial increase due to the incorporation of the four disruptions into the same model. The average minimum cost for the Monte Carlo simulation is 1,553,475,313 PKR, compared to the deterministic model’s 1,067,342,436 PKR, reflecting a 45% increase. Figure 3 illustrates the trend for the minimum cost at optimum flows between entities. Initially, the value is at its peak in scenario 1, but as the model progresses, the overall trend begins to decline. Most total costs remain within an interval of 1 × 10^9^ PKR of each other. The convergence of the values illustrates that the model gradually tends towards stability and would show less deviation if the number of scenarios is increased. In Monte Carlo method, the converging values mean that the estimate’s variability decreases as more trials are run. In other words, the simulation is producing increasingly good approximations of the true underlying value or distribution.

In the later scenarios, it becomes evident that the variation is diminishing, and values are converging, indicating that the model is gradually approaching a point with minimal change. Table 7 showcases the output from refineries, which, as expected, demonstrates that the overall flow of refined products sourced from refineries is less than that in the deterministic model. The volume flow by road and rail has increased due to the disruption caused by the non-functional white oil pipeline.

**Table 7:** Transportation volumes (in Tons) –- refineries to Depots.

Refineries	Depots					
	Road (HSD)	Road (PMG)	Rail (HSD)	Rail (PMG)	Pipe (HSD)	Pipe (PMG)
1	300,644	-	-	-	645,374	1,246,122
2	-	-	732,627	566,876	468,127	-
3	-	50,000	-	-	1,369,851	716,812
4	2,175,123	1,543,641	337,999	913,326	1,227,575	-
5	498,762	173,547	-	-	906,060	330,833
Total	2,974,529	1,767,188	1,070,626	1,480,202	4,616,986	2,293,767

Transshipment between storage facilities has also increased, particularly by road. This transshipment is critical since there are no pipelines and railway tracks directly linking the ports with storage facilities in the north. However, due to the absence of a railway network between storage facilities, this could not be fully implemented. For both HSD and PMG, pipeline volumes have more than doubled, around 243% and 251% respectively, owing to the higher demand for fuel and increased transportation costs via other modes. The results are depicted in Table 8.

**Table 8:** Transshipment volumes (in Tons) between Depots.

Depots	Transshipment Volumes (Road, Rail, Pipe)					
	Road (HSD)	Road (PMG)	Rail (HSD)	Rail (PMG)	Pipe (HSD)	Pipe (PMG)
HSD PMG	1,204,898 -	3,497,183 -	- -	- -	2,740,518 -	10,329,836 -

Regarding imports, both products have witnessed an increase of over five times their deterministic values. The shortage of refined products from local refineries, coupled with the surge in the use of petroleum products, has led to an escalation in imports to meet the demand. Table 9 illustrates the utilization of both import terminals, with PMG being primarily imported to meet the requirements of the transport industry. HSD was previously not being imported through Port 1 but is now, along with a 65% increase in volume by Port 2. PMG imports also increased at both locations by 86% and 73%, respectively.

**Table 9:** Transportation volumes (in Tons) –- ports to Depots.

Ports	Transshipment Volumes (Pipe)		% Change vs. Deterministic value	
	HSD	PMG	HSD	PMG
1	2,747,850	12,190,087		86
2	2,893,636	4,765,833	65	73

**Fig. 4:**
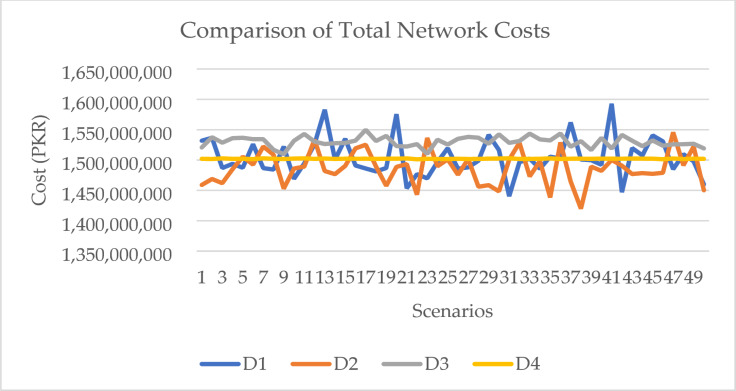
Comparison of Scenario wise minimized network transport cost (PKR).

The total network cost comparison between disruptions is given in Fig. 4. The costs associated with all disruption scenarios are higher than the cost calculated of the deterministic model. Disruptions caused by increase in demand for fuel caused the most increase in the overall supply chain cost. Demand for both fuels increased due to the economic recovery efforts after the COVID-19 pandemic. The rise in demand led to more volume being procured, stored and transported, which increased the operational cost of the entire supply chain. Transport cost is the highest contributor as all of the movement in the second echelon of the supply chain is done by road. Both, pipeline and rail, transport modes also show an increase in volume flows as well. Volume flows from refineries and import terminals are also increased to fulfil the demand requirements.

Disruptions on the supply side caused the second most increase in overall network cost. The reason for this is because the local refineries were not able to provide enough products to the high demand regions, which ultimately led to procurement of imported fuel. The distance between the high demand regions and import terminals added to the increase in total network costs as the fuel had to be transshipped across the country to areas which could have been easily supplied by refineries, which in this case were facing production issues.

Increase in transport cost impacted the supply chain network to a lesser extent compared to supply and demand side disruptions. All transport modes showed an increase in the cost to move refined fuel products. The highest increase in cost per unit volume was of road carriages, followed by pipeline, and then railway carriage. The impact of this disruption shifted to the flow of products towards the pipeline mode. The route which connected multiple storage facilities was more prominent as it allowed for cross country movement at the least operational cost. In the first stage of the supply chain, refineries also increased rail carriage flows whenever the infrastructure was available. Due to lack of any other transport mode available, the last stage of the supply chain is solely serviced by road carriages. The cost associated with supplies to customer nodes is the largest contributor to the increase in total network cost due to multiple customer nodes being supplied by a single storage facility and the distance between the two entities.

Bulk oil pipeline disruptions caused the least impact on the total supply chain network costs. The white oil pipeline connecting storage facilities, near import terminals, with the storage facilities in the north of the country were not operating. This created an unanticipated supply chain disruption by reducing the flow of products towards the high demand regions. The supply chain was least affected by this disruption due to availability of other transport modes which were able to absorb the volume otherwise moved through the pipeline. The cost increase was due to the movement of fuel by road carriages. Transshipment also increased as road movement is limited to only between storage facilities that are near to each other. Railway movement was not used due to lack of proper infrastructure.

The overall analysis of the multiple disruption scenarios shows that the increase in demand affects the total supply chain cost the most, followed by reduction in supplies by the local refineries. Increase in transport costs along with any operational disruptions affect the total network costs to a lesser degree. This is because of presence of substitute services that can be utilized to mitigate the effects of these disruptions. Transport modes compete with each other on the basis of price, availability and reliability. Any issue with one mode creates an opportunity for the remaining modes to cover up for the volume lost. This is not possible for supply and demand side disruptions as there are no substitutes for the refined fuel products and therefore are most sensitive to market forces. Mitigation strategies are only limited to alternate sourcing and increase in inventory capabilities, which also increase operational costs.

## Conclusion and future perspective

The aim of this research is to develop a MILP model that optimizes a multi-stage, multi-product, and multi-modal downstream petroleum supply chain network of a company in an oil-importing country. Specifically, the study focuses on two products, namely, PMG and HSD. The research is divided into two key stages: a deterministic model that establishes the baseline operating conditions of the supply chain in the absence of disruptions, and a Monte Carlo simulation of the same model under different disruption scenarios. The purpose is to comprehend the volume flow of products between entities and the overall impact of disruptions on the entire supply chain network cost. The disruption scenarios exhibit variations in terms of the parameters they impact, and they are all considered simultaneously to replicate a situation in which multiple disruptions can occur concurrently. The comparison is made between the deterministic model and the average volume flows between entities across all scenarios. The findings underscore the importance of strategic supply chain planning and tactical operations to minimize the overall transport cost. The simulation spans a “1-day” time period, where the parameters are set to reflect the daily averages in the oil industry^31^.

The results reveal that concurrent disruptions can potentially increase the overall network transport cost by as much as 50%. Issues in the production of refined oil by refineries lead to a decrease in volume movement from refineries to storage facilities. Furthermore, an increase in customer demand results in a rise in the volume flow from storage facilities. Both disruptions accentuate the necessity for increased imported fuels to counter the effects of supply shortages and meet the overall demand^32^.

The third disruption examines the effect of rising business costs for the company, as the transport cost per unit escalates across all modes. Consequently, more volume has to be transported through pipelines to make the supply chain more cost-effective, necessitating strategic supply to satisfy high-demand regions such as the north-east. Lastly, a structural disruption in the form of a pipeline shutdown is analyzed, compelling the company to shift its transportation operations to the road. This leads to an overall increase in transshipment between storage facilities, reducing the distances traveled by oil tankers in each journey^33^.

The results of this research can be beneficial to design robust oil supply chains that can resist random and anticipated disruptions to a better extent, especially in countries that do not have widespread developed infrastructure. Regions that are net oil importers are most vulnerable to global fluctuations and are therefore negatively affected.

### Implications and future research

From managerial perspective, the insights suggest that logistics should be designed with priority towards flexible transport strategies, such as building redundancy in pipelines and ensuring multimodal access. Distributors and refineries should also factor in potential need for short-term sourcing through imports when refinery outputs face reductions. The findings also highlight policy reformation for governments, particularly for oil importing nations. Investment in resilient infrastructure such as pipelines and shared storage depots should be incentivized. A regulatory framework that enables companies to cooperate during crisis should be created that can reduce systemic vulnerabilities. A broader takeaway is making DOSC resilient to disruptions may not necessarily need monumental efforts but rather adaptive adjustments to already existing infrastructure and facilities. This insight is particularly relevant to developing countries with limited infrastructure, where resilience strategies must be both cost effective and scalable.

For future studies, it is recommended to simulate the effects of these disruptions on depot inventory volumes. Additionally, the research could explore how these effects can be minimized by implementing structural and strategic improvements that can be replicated in other similar markets. Vehicle capacities were also not considered as an additional constraint. The vehicle capacity assignment can be modeled with a separate transportation linear programming model. This is because vehicles have multiple options in terms of capacity, and their use depends on whether they supply one node or multiple nodes. Moreover, the addition of operational and capital costs can be added to make a more comprehensive model.

## Data Availability

The datasets used and/or analyzed during the current study are available from the corresponding author on reasonable request.
